# Combined Dielectrophoresis and Impedance Systems for Bacteria Analysis in Microfluidic On-Chip Platforms

**DOI:** 10.3390/s16091514

**Published:** 2016-09-16

**Authors:** Cristina Páez-Avilés, Esteve Juanola-Feliu, Jaime Punter-Villagrasa, Beatriz del Moral Zamora, Antoni Homs-Corbera, Jordi Colomer-Farrarons, Pere Lluís Miribel-Català, Josep Samitier

**Affiliations:** 1 Department of Electronics, Bioelectronics and Nanobioengineering Research Group (SIC-BIO), University of Barcelona, Martí i Franquès 1, 08028 Barcelona, Spain; ejuanola@el.ub.edu (E.J.-F.); jpunter@el.ub.edu (J.P.-V.); bdelmoral@el.ub.edu (B.d.M.Z.); antoni.homs@icn2.cat (A.H.-C.); jcolomer@el.ub.edu (J.C.-F.); pmiribel@el.ub.edu (P.L.M.-C.); jsamitier@ibecbarcelona.eu (J.S.); 2 IBEC-Institute of Bioengineering of Catalonia, Nanobioengineering Research Group, Baldiri Reixac 10-12, 08028 Barcelona, Spain; 3 CIBER-BBN-Biomedical Research Networking Centre for Bioengineering, Biomaterials and Nanomedicine, María de Luna 11, Edificio CEEI, 50018 Zaragoza, Spain

**Keywords:** dielectrophoresis, impedance, bacteria, on-chip, microfluidics

## Abstract

Bacteria concentration and detection is time-consuming in regular microbiology procedures aimed to facilitate the detection and analysis of these cells at very low concentrations. Traditional methods are effective but often require several days to complete. This scenario results in low bioanalytical and diagnostic methodologies with associated increased costs and complexity. In recent years, the exploitation of the intrinsic electrical properties of cells has emerged as an appealing alternative approach for concentrating and detecting bacteria. The combination of dielectrophoresis (DEP) and impedance analysis (IA) in microfluidic on-chip platforms could be key to develop rapid, accurate, portable, simple-to-use and cost-effective microfluidic devices with a promising impact in medicine, public health, agricultural, food control and environmental areas. The present document reviews recent DEP and IA combined approaches and the latest relevant improvements focusing on bacteria concentration and detection, including selectivity, sensitivity, detection time, and conductivity variation enhancements. Furthermore, this review analyses future trends and challenges which need to be addressed in order to successfully commercialize these platforms resulting in an adequate social return of public-funded investments.

## 1. Introduction

Bacteria-related diseases caused by ingestion of contaminated food or water result in considerable morbidity and mortality representing a significant public health threat in developed and developing countries [1,2]. In the United States 3000 fatalities caused by food-borne infections were reported in 2012, and in 2013, 11,000 infections were recorded for the same cause [3]. Each year, there are more than 2.5 million deaths due to water-associated diseases worldwide [2,4]. In this context, diagnostic devices are extremely important for implementing an effective response to the prevention of bacteria related diseases [5,6], water treatment [7], and public health [8], preventing millions of deaths caused by the lack of these facilities [9].

Numerous methods exist to mitigate these issues based on the separation and concentration of bacteria (see Appendix) [10]. Traditionally, this is performed in the laboratory and using commercial equipment [11]. Conventional pathogen detection methods include metabolic tests based on media, the use of enzyme-linked immunosorbents or pathogen-specific antibodies coated into magnetic beads, and oligonucleotide arrays for amplifying hybridized DNA fragments of bacteria. Some of the approaches to concentrate bacteria take advantage of the different properties of the cells. For example, physical properties are being exploited by techniques such as centrifugation or filtration [6]. Mass spectrometry (MS) and capillary electrophoresis (CE) take advantage of chemical or electrodynamic properties [12,13]. Other methods for separate and concentrate bacteria are based on immunological approaches such as immune separation [6] and the enzyme-linked immunosorbent assay (ELISA) [14]. Microscopy advances such as fluorescence or Raman microprobe spectroscopy (RMS) [15,16] are also used. Others are nucleic acid probe-based such as the ligase chain reaction (LCR) [17], microarrays and Polymerase Chain Reaction (PCR) [18,19].

These diagnostic tools are elaborate and expensive because of the equipment and time (typically demanding several days) [20]. In particular, current methods require more than 5–7 days for identification of pathogenic bacteria [14]. In addition, the majority of them are not portable, prevention of contamination is difficult due to the small volumes, becoming a challenge to concentrate the bacteria in a microlitre or even nanolitre sample, and, in most cases, alternative methods require operation with a reagent, so the posterior bacteria detection process is rather complicated [21]. As an aggravating factor, the heterogeneity of individual cells makes these methods unsuitable for all kinds of bacteria [11,20].

The criteria recommended by the World Health Organization says that infectious disease diagnostic platforms must be specific, sensitive, simple-to-use, accurate, rapid, low-cost and robust [22,23]. There have been important attempts to accomplish these requirements, especially for laboratories interested in creating novel microfabricated structures for other specific uses [24]. However, even though there have been many published studies during these last two decades [5], few outcomes of microfabrication technologies have been successfully introduced onto the market (such as lab-on-a-chip (LOC) devices) [22,25,26]. Examples include the Immunocard STAT (Meridian Diagnostics, Cincinnati, OH, USA), which is a portable system and fast test for detecting *Escherichia coli* (*E. coli*) O157+H7 in faeces [18,27]. This kit has a high sensitivity (87%) and specificity (97%), however it cannot detect non-O157 STEC serogroups [28]. 

Some other examples include the *Mycobacterium Tuberculosis* Direct Test (MTD) from Gen-Probe (San Diego, CA, USA), the Probe Tec ET (BD, Franklin Lakes, NJ, USA) and the COBAS AMPLICOR (Roche, Pleasanton, CA, USA) devices for mycobacterial detection [18].

Despite the portability and highly-sensitivity advantages of these artefacts, not all of them meet time and cost needs. This generates an urgent necessity for fast, accurate, cost effective and more accessible technologies [25]. Due to this scenario, new methods of fast monitoring and characterization have been explored based on electrical properties of cells or particles [29,30]. In this context, electric field-based separation approaches are attracting interest because of their fastness, potential for automation, simplicity, portability, miniaturization, massive parallelization and labour-saving characteristics [10,11,31]. Based on their distinct electrical properties, dielectrophoresis (DEP) is a versatile technique used for the rapid detection and separation of particles. Even this technique was initially discovered by Pohl and colleagues in the 1950s [32], it has developed an exponential booming in the last fifteen years [33,34]. 

**Figure 1:**
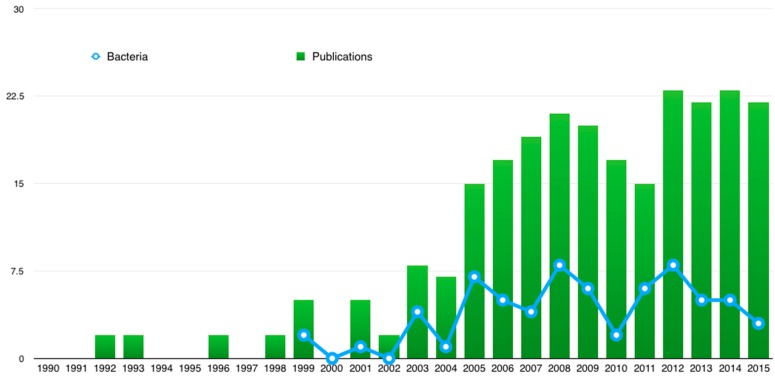
Publishing trends with “dielectrophoresis” and “impedance” keywords in Web of Science from 1990 to 2015. Blue line indicates the same keywords plus “bacteria”.

An effective strategy to enhance sensitivity in a reduced detection period is by combining DEP with impedance analysis (IA) [35]. DEP and IA coupling has emerged in recent years. This can be evidenced in the growing number of published articles and citations reflected in Web of Science (WOS). This emergent trend is also evident for bacteria detection and concentration (Figure 1) since several research groups reported the simultaneous measure of the concentrated bacteria in a single piece of equipment [14,30,35,36,37,38]. 

**Figure 2:**
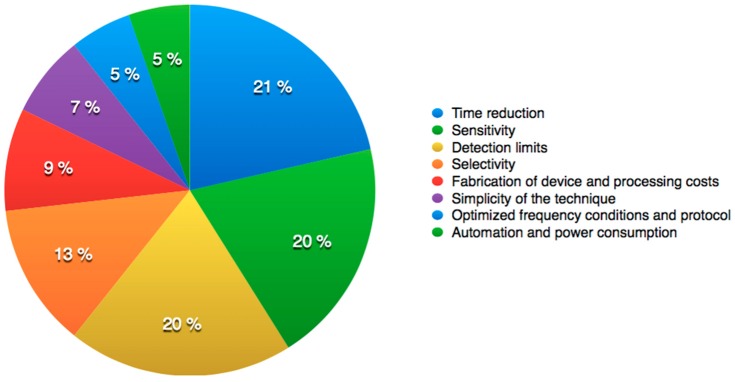
Addressed technical challenges that combined DEP and IA for bacteria analysis found in WOS publications from 1990 to 2015.

The advantages of the combined method have prompted researchers to improve some technical aspects to overcome some of the challenges that are inherent from bacteria. In this context, numerous aspects related to manipulate, select and quantify bacteria have been improved over the years. Some of these aspects include both device and protocol optimization (Figure 2). We found that in publications where DEP and IA are combined for bacteria analysis, improvements related to selectivity, sensitivity, and detection times are the most studied challenges. Due to this scenario, and taking into account future challenges to take into consideration, authors find it important to analyse approaches from recent studies that share the same needs and goals when DEP and IA are being combined.

This document reviews the state-of-the-art approaches that take advantage of these two technologies focusing on bacteria concentration and detection, independently of their original growth medium. The aim is to analyse the challenges overcome and the principal opportunities that are facing LOC devices in a technology convergent scenario focusing on the emerging trend of microfabrication for envisaged LOC devices. It is necessary to review this combined approach, which can have a great impact in numerous fields such as medicine, biology, agriculture and environment [18,39,40]. 

The following Section 2 introduces the concept and applications of these two methods and reviews recent approaches using DEP and IA for bacteria concentration and detection. Next, in Section 3, some of the relevant operational improvements of recent studies are analysed. Section 4 describes future considerations and challenges to be taken into account for the commercialization of emerging DEP and IA micro-devices. Section 5 analyses the innovation and technology transfer aspects that these devices require for reducing the gap between research and society. Finally, in Section 6 we present the conclusions of this review.

## 2. Theoretical Background

### 2.1. Dielectrophoresis (DEP)

DEP is one of the currently used strategies in microfluidics for a versatile and label-free detection and separation of particles based on their distinct electrical properties [41]. It is described as the physical phenomenon whereby neutral particles move when a non-uniform electric field is applied according to the particles and medium physical properties [39,42,43]. The permittivity, conductivity, and dielectric properties determine the translational motion of the particle [44]. DEP uses a nontoxic electrical stimulation to induce a frequency-dependent dipole in cells [45]. The dielectrophoretic force is defined by Equation (1) [36,46,47]:

(1) $$F=2\pi \varepsilon_{m}\;R^{3}\;\text{Re}\;\left[\underline{CM}\;\left(\omega \right)\;\nabla {\underline{E}}^{2}\;\left(r,\;\omega \right)\right]$$

where **F** concerns to the dipole approximation to the DEP force, $\varepsilon_{m}$ refers to the permittivity of the medium surrounding the sphere, *ω* is the radian frequency of the applied field, *R* corresponds to the radius of the particle, **r** is the spatial coordinate, and **E** refers to the complex applied electric field. *CM* is the Clausius-Mossotti (CM) factor that is given by:

(2) $$\underline{CM}=\frac{{\underline{\varepsilon }}_{2}-{\underline{\varepsilon }}_{1}}{{\underline{\varepsilon }}_{2}+2{\underline{\varepsilon }}_{1}},$$

where ${\underline{\varepsilon }}_{1}$ and ${\underline{\varepsilon }}_{2}$ are the complex permittivities of the medium and the particle, respectively, and are each given by $\underline{\varepsilon }$ = ε + *σ*/(*jω*), where *σ* is the conductivity of the medium or particle, ε is the permittivity of the medium or particle, , and *j* is $\sqrt{-1}$. The sign (+/−) of the CM factor determines a positive DEP (pDEP) if the DEP force propels particles toward the electric-field maxima, or a negative DEP (nDEP) if the force propels particles toward the electric-field minima.

The wide range of capabilities enabled through the DEP technique include concentrating [21], sorting [48], rotating [49] and moving particles or biological material [50,51]. Studies have demonstrated that DEP is a promising technique for bacterial concentration with potential biosensor applications [40,52,53] since it allows the advanced multifunctional and rapid detection of micro-organisms at lower flow rates and bacteria losses [54,55]. These capabilities are not only exclusive for bacteria but also for DNA [56], proteins higher than 105 Da [42], cancer cells [57], foetal nucleated red blood cells, thrombloplasts [58], red/white blood cells [59], yeasts [60,61,62], viruses [63,64,65] and particles such as carbon nanotubes [66] and submicron particles [67]. 

Although DEP offers several advantages over other methods it has some limitations. Bacteria, as well as other single cell organisms, respond to their surroundings and media. Particle effects can be sensitive to the parameters of the medium such as pH, conductivity, temperature and electrolyte valency. Additionally, the particle surface can absorb reagents present in the medium [68]. Therefore, these external factors must be controlled and consistent harvest concentrations and methods should be used from cultured cells in order to have consistent DEP results [69]. Moreover, it is important to previously modify the surface charge before changes in DEP behaviour. Another difficulty is the integration of DEP into miniaturized systems. This challenge is primarily due to complex electronic control architectures, and the incompatibility with heterogeneous sample matrices [70]. 

### 2.2. Impedance (IA)

IA is an electrochemical technique that provides information on bio-affinity-event induced changes in resistance and capacitance at the surface of a substrate or electrode [71]. Impedance analysis (IA) is related to electrical properties of particles. The impedance from each partial circuit and the total impedance were defined by [72] in the following equations:

(3) $$\frac{1}{\left|Z\right|}=\frac{1}{\left|Z_{1}\right|}+\frac{1}{\left|Z_{2}\right|}$$

(4) $$\left|Z_{1}\right|=\sqrt{R_{sol}^{2}+\frac{1}{{\left(\pi \;f\;C_{dt}\right)}^{2}}}$$

(5) $$\left|Z_{2}\right|=\frac{1}{2\pi \;f\;C_{de}}$$

where *f* represents the excitation frequency, *R_sol_* the solution resistance, *C_dt_* the double layer capacitance, Z_1_ the impedance of the *R_sol_* and *C_dt_* , *C_de_* the solution dielectric capacitance and Z_2_ is the impedance of *C_de_*. Z is the total impedance of the parallel Z_1_ and Z_2_, as shown in Equations (4) and (5) [72].

Impedance frequency dependence, has been demonstrated to be efficient for characterizing cells and their comportment both in nano-, micro- and macro-fluidic systems [73,74], therefore, this label-free technique is applied in many biological fields for biochemical concentration measurements [71,75,76]. Even though impedance detection is simple to design, and has high sensitivity and detection limits [77] the accurate measurement of biophysical properties of cells in microfluidic devices is limited by the high impedance of probe electrodes, the electric double layer and stray capacitance [78]. 

Impedance measurements are largely used in LOC devices to detect antibodies, virus, receptors, enzymes, DNA or many cell types (macrophages, endothelial cells, blood cells, fibroblasts, etc.) [35,74,78,79,80,81,82,83,84,85]. Single cell IA also resulted in an effective method for cell counting, discrimination, behaviour analysis and growth of bacteria [35,86,87]. *Impedance microbiology* measures the variations in electrical impedance of a culture medium or a reactive solution that results from the bacterial growth [55,88]. Previous studies have reported the use of this technique to detect and quantify different species of bacteria [14,89,90] such as *Salmonella* [91,92,93], *E. coli* [94,95], *Listeria innocua* and *Listeria monocytogenes* [96], *Staphylococcus aureus* [97], *Enterococcus faeccalis* [98], coliforms, *Listeria* spp., and *L. monocytogenes* [55]. Detection times ranging from 24 hours [99] to seconds [100] have been reported.

### 2.3. The Combined Approach for Bacteria Concentration and Detection

**Figure 3:**
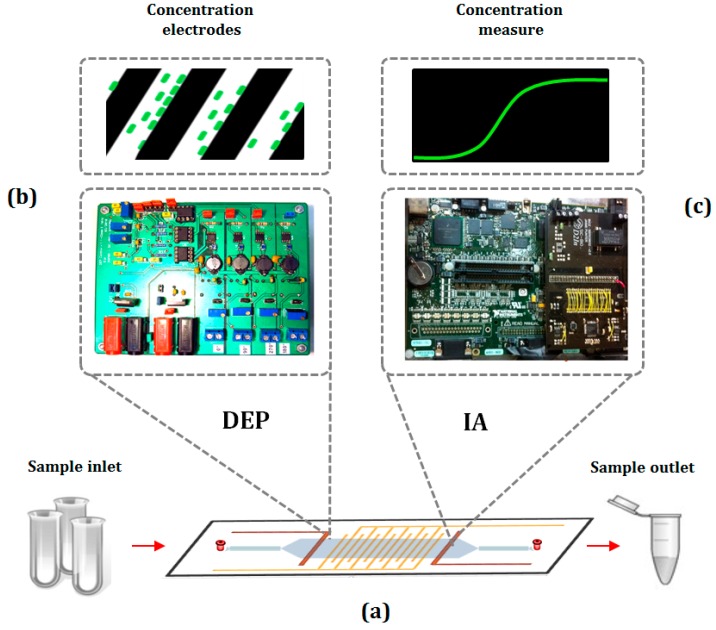
Scheme of the overall process. (**a**) The electronic module; (**b**) Bacteria concentration by dielectrophoresis; (**c**) Concentration measure by impedance analysis (adapted from [36]).

Currently, some biosensors are capable of combining DEP and IA on a microfluidic chip. These chips are devices usually comprised of a LOC and a customized electronic unit (Figure 3a). The DEP force pre-concentrates the sample in this electronic unit (Figure 3b) and IA monitors this concentrated sample (Figure 3c). DEP modifies the low-frequency capacitance (<100 kHz) due to particle concentration on the electrodes, as the cells are trapped by the DEP force at the interdigitated array microelectrodes (IDAM), its permittivity substitute an equivalent volume of the medium. In consequence, the impedance among the electrodes will change with the variations in the complex permittivity of the medium that divides them and this can be plotted in a graph [44]. At high frequency ranges, the electrical signal applied to measure the impedance flows through the inner cell, reporting information about the inner cell properties, and it is better used for single-cell cytometry.

The combination of DEP and IA has demonstrated to be effective for the detection of DNA [101,102], RNA [100], yeasts [59,103], virus [104], cell trapping, detection and lysis [105,106], cancerous cells [107,108,109,110], and for bacteria [35,36,38,76,90,111,112,113,114,115,116,117,118]. Some of the devices used in bacteria concentration and detection are summarized in Table 1.

### 2.4. Recent Approaches

One of the first approaches combining DEP and IA, was developed by [119]. This group of researchers studied the quantitative estimation of *E. coli* in an aqueous medium by applying positive DEP (pDEP), which occurs when the cell is attracted to the electrical field maximum. The time required for detection was 10 min. In the same year, viable and non-viable *E. coli* were selectively detected by [38] by studying the effects of viability and sterilization on DEP and impedance measurements. Bacteria trapping was tested by using different frequencies (100 kHz and 1 MHz). By applying 1 MHz of electrical field, they selectively collected viable and heat-sterilized non-viable bacteria by pDEP and sensed them by DEP and IA. They argued that heat treatment is the responsible of the change of the dielectric properties of cells, showing a decrease in the cytoplasmic conductivity.

Two years later, higher sensitivity for bacteria detection was achieved by incorporating electropermeabilization (EP). EP is the implementation of a strong electric field in order to increase membrane permeability. If the membrane is permeable, intracellular ions are liberated and disseminated into the external medium acting as ionic current carriers. This increases the conductance and avoids electrolytic contamination produced by metal ions. They finally obtained a concentration of bacteria of 10^4^−10^2^ CFU/mL after 3 h of experimentation [116].

**Figure 4:**
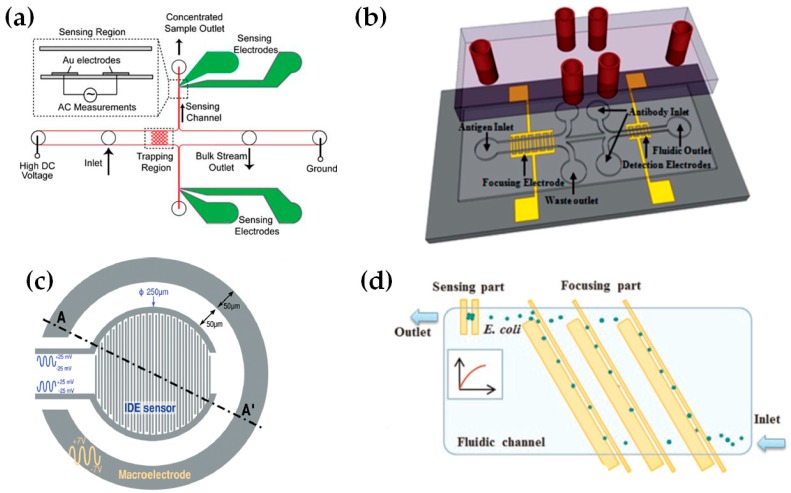
Schematic of bacteria concentration and detection approaches using DEP and AI. (**a**) This device selectively concentrates pathogens on the base of their size by DEP and high DC voltage. The concentrated sample is released for the measure of AC impedance by a pressure-driven flow [46]; (**b**) Design of a DEP and IA device with two IDAMs in a SU-8 microchannel [14]; (**c**) A device containing a IDAM (for capacitive sensing) and a macroelectrode (for electrokinetics). A cross.section of the AA’ plane [37]; (**d**) Design of the sensor consisting of a pDEP region and a sensing region that employs dielectrophoretic impedance measurements [30].

Another study focused on the enrichment of bacteria was developed by [46]. This was the first study reporting the implementation of insulator-based dielectroforesis (iDEP) and IA for *B. subtilis* concentration and detection. iDEP is a technique adapted from DEP which provides an insulating layer on the top of the electrodes to protect them, and where the substrate material is the only material which is in contact with the sample [37,120,121]. The possibility of linking iDEP with impedance detection resulted in trustworthy enrichment of particles. With this approach they also demonstrated that impedance detection is dependent on the signal frequency and particle concentration (Figure 4a).

Alternatively, [35] doubled the sensitivity of *E. coli* detection by implementing negative DEP (nDEP) before applying pDEP and impedance for detection. In nDEP, particles are attracted to an electrical field minimum. They used a device composed of two microelectrodes. The first microelectrode was used for bacteria concentration using nDEP energized with 1 kHz frequency. The second was used for bacteria detection by pDEP energized with 100 kHz. The different voltage values were determined through a theoretical prediction in order to know at what frequencies nDEP or pDEP occurs. Their approach is useful to reduce the longer detection periods often required for low bacterial concentration samples where it is necessary to trap a large number of cells.

In 2013, Dastider and collaborators developed an impedance biosensor for detecting of *E. coli* O157:H7 that also improved measurement sensitivity by using pDEP and two sets of gold IDAM (Figure 4b). Initially, positive electrophoresis was used to focus and concentrate the bacteria in a microchannel in the first set of IDAMs and the second set was used for impedance measurements. Their lowest limit of detection (LoD) was 3 × 10^2^ CFU/mL within a preparation time of more than 1 h [14]. 

More recently, another approach aimed at increasing the sensitivity of the device is reported by [37]. They developed a device that combines a circular shaped IDAM, with a surrounding macroelectrode. These allowed a higher sensitivity surface sensing and volume in order to trap bacterial cells by incorporating AC-electro-osmosis (AC-EO) (Figure 4c). Their device demonstrated that the LoD can be reduced from 3.8 × 10^6^ CFU/mL to 3.5 × 10^5^ CFU/mL by applying this electrohydrodynamic effect in a whole-cell *Staphylococci epidermidis* after 20 min of incubation. This LoD reduction is due to the fluid flow generated by AC-EO that causes indirect bacterial motion, improving the sensitivity of detection. Again, these types of devices are necessary for low bacterial concentrations. However, based on their detection time, they are not adequate at emergent sanitary conditions.

In this context, different solutions and approaches have been reported, such as [30,31]. [30] developed a device capable of detecting bacteria in 1 min. This was performed in drinking water for *E. coli* (Figure 4d). They used pDEP since drinking water’s low conductivity makes it difficult to analyse by nDEP. In this study two electrode widths (100 and 30 µm) were configured for a bacteria flow rate of 1500 µL/h. Also, they determined that the optimal detection limit is 300 CFU/mL across different populations examined (150, 300, 750, and 1500 CFU/mL). 

A more rapid and continuous flow microfluidic chip was developed by [36] capable of injecting, trapping, cleaning and continuously measuring impedance every 30 s. The device was capable of concentrating 2 × 10^7^ cells/mL of *E. coli* 5K strains at several continuous flows (5 to 30 µL/min) with the utilization of pole structures, and 44.2% less bacteria losses. 

All of these contributions showed that DEP and IA for bacteria concentration and detection is being enhanced in various ways, namely, LoD, sensitivity and detection times. This last point for example, has been reduced from hours to minutes. Additionally, they are not exclusive to one species of bacteria. In this regard, there has been much progress concerning selectivity, conductivity variations and flow conditions, involving advances in such different technologies as microfluidic design, microstructure engineering, electronic instrumentation, and computational data processing. These improvements are addressed in the following section.

## 3. Operational Improvements of Combined DEP and IA Targeting Bacteria

### 3.1. Selectivity and Sensitivity

Methods for detecting bacterial have the imperative necessity to be selective and sensitive due to the few number of bacteria present in a sample [77]. Even more, when pathogenic bacteria is often present with non-pathogenic ones [122]. However, the accurate measurement of biophysical properties of cells in microfluidic devices is limited by the high impedance of probe electrodes, the electric double layer and stray capacitance [78]. 

Some of the approaches to improve detection selectivity when combining DEP and IA take advantage of the agglutination phenomenon caused by the antigen-antibody bonding. This bonding allows immobilization of the bacteria on the device [30,123] according to their viability or species type [122]. The immobilized antibodies and the target bacteria banded to the electrode change the electrochemical impedance, detecting the target bacteria and measuring the impedance of the antibody [35]. After voltage is applied and turned off, the sample solution is washed away, excluding the target bacteria. Bacterial cells can conduct when they are present in between two conductors in an IDAM array because it cell wall, cytoplasm and few other cell components act as conductors [124]. Then the bacteria could be identified and quantified by quantifying the electrode’s residual impedance [35]. 

According to [38], there are two methods of using antigen-antibody reaction for bacteria selection. The first one consists in adding the antibody to the cell suspension for the agglutination of the antibody-specific bacteria after DEP enrichment. The second method consists in immobilizing the antibodies onto the microelectrode before DEP, in order to bound the immobilized antibody into the antibody-specific bacteria. 

Undesired non-specific bacteria binding still occurs even using this antibody-modified chip [122] and the bio-recognition component can be a disadvantage [77]. Moreover, polyclonal antibodies used as the bio-affinity element to characterise the bacteria require consumption of reagents, increasing costs and detection times [24].

Improved methods for bacteria selectivity are not exclusive of vegetative forms but also to sporulated forms. Characterization of this structure is not easy because dormant cells are not actively generating considerable levels of metabolites. However, bacterial spores have great interest, for example, for *Bacillus anthracis*. [125] have demonstrated that spores selectivity could be achieved by combining DEP and IA. By testing over a mixture of *B. mycoides* and *B. subtillis* spores, they showed that the electrical response of a spore in a gap between two planar microelectrodes can discriminate between different species and subspecies of *Baccillus*. In presence of an electrical potential, the surface charges, responsible of the hydrophilicity of spores, serve as charge carriers. The character of this surface charge explains the species-specific variations in hydrophobicity and impedance too.

Spore selectivity can be improved by using fluorescent polystyrene beads in order to eliminate particles of interest. [46] demonstrated this improvement in *B. subtillis* spores. They injected fluorescent polystyrene beads with 2 μm of diameter into a microchannel (10 μL/min of injection rate). The resulting scenario showed that only one particle type can be selectively concentrated and diverted down the side channel, allowing the approximation of the concentration of the particles by impedance measurements. Contaminants are putting apart or reduced facilitating the detection only of the particles of interest. The use of fluorescent polystyrene beads can be extent to nano-sized particle detection [126], however, prior labelling requirements can be a drawback of this technique [127].

### 3.2. Fouling

**Figure 5:**
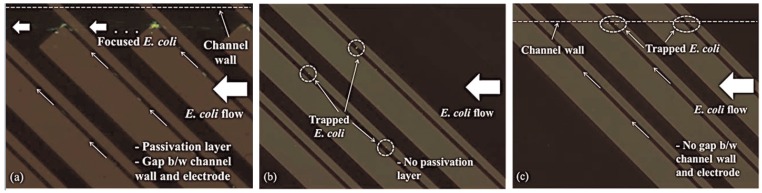
Characterization of pDEP-based *E. coli* focusing. (**a**) The electrode is covered by a passivation layer. Cells flow through the electrode edge and are liberated at the end of the electrode; (**b**) Cells are not flowing. They persist trapped on the electrode, which is not covered by a passivation layer; (**c**) Cells flow along the electrode but not liberated from it (reproduced with permission from [30]).

On the other hand, label-free approaches have demonstrated to improve other operational challenges such as fouling (the adhesion of cells to the electrode edge), electrode delamination or bubble formation. [30] used iDEP, also known as contactless DEP (cDEP), with IA using a passivation layer on the electrode to permit efficient bacteria focusing under high flow conditions. In this study, they also demonstrated that the geometry and disposition of electrodes play an important role in cDEP since a decreased electrode width increased the sensitivity of the sensor. They evaluated several types of electrodes tested under same experimental conditions for *E. coli* and showed that a gap among the electrode edge and the channel wall, as well as the passivation layer used were crucial for effective DEP focusing. This phenomenon could be seen at the Figure 5, which depicts the motion of *E. coli* in the focusing electrode. Due to the round shape of the electrode edge, the bacteria were liberated at the end of the electrode. Figure 5b shows the control experiments with no passivation layer and Figure 5c using passivation layer without a gap between the channel wall and the electrode edge. In both, the high pDEP force caused the incapability of *E. coli* to flow along the electrode edge.

This technique has some drawbacks. First, the use of the passivation layer requires special attention in order to achieve successful focusing and sensing. For instance, a high electric field could reduce the layer lifetime [30]. Second, joule heating and an increasing of temperature is caused by the highly conductive biological fluid and the high electric field intensity [120]. Additionally, manipulating particles and cells is difficult with iDEP and cDEP due to the collecting patterns, confirming this is still challenging [128].

Rather than using patterned surface electrodes, an electrically conductive liquid metal used as the electrode can be controlled. This improvement refers to the concept of *liquid electrodes* initially developed by [129,130]. Electrodes constitute a very important element in these systems but their implementation has some disadvantages. First, they require complicated fabrication procedures [120,131]. Second, they are susceptible to suffer from fouling, bubbles, and low throughput [120]. Liquid electrodes are recessed electrodes positioned perpendicularly to the main channel. Electrodes are then polarized by inverted signals in order to generate the lateral DEP force necessary for manipulation of particles in the main channel [132]. The result is to a homogeneous electrical field over the total channel [130]. Even these electrodes improve the spatial resolution and increases the resolution range with a simplified fabrication process and reduced costs, it has been shown that decreases the sensitivity compared to top-bottom electrodes [133]. 

### 3.3. Buffer Conductivity Variations

On the other hand, another very critical problem in impedance measurement involving bacterial species is the buffer conductivity. Buffer is the liquid where cells are suspended, independently of its origin and/or composition, and this is considered as our media. There is a governing effect of sample conductivity variations on the impedance quantifications when this media is not controlled [77]. The cellular solution conductivity changes through time, and produces a masking effect on the impedance measurements. Therefore the quantified impedance is totally dependent to sample buffer conductivity, and not to the concentration of bacteria [77,134].

Only one previous study has confronted conductivity variations. [36] developed a device in which the variation of the conductivity was corrected through a specially designed automated protocol, composed of media conductivity stabilisation and DEP voltage disconnection during impedance measuring. On this study, the conductivity of the media linearly increased from 8.2 × 10^−5^ S/m to 2.5 × 10^−3^ S/m. The stabilisation was achieved by controlling buffer conductivity using Milli-Q water. Impedance changes are highly associated to variations in the conductivity of the media due to bacteria when cleaning processes does not control the cells’ media. Therefore, for ensuring a reliable measurement, it was implemented an automatized and periodic cleaning process. 

**Figure 6:**
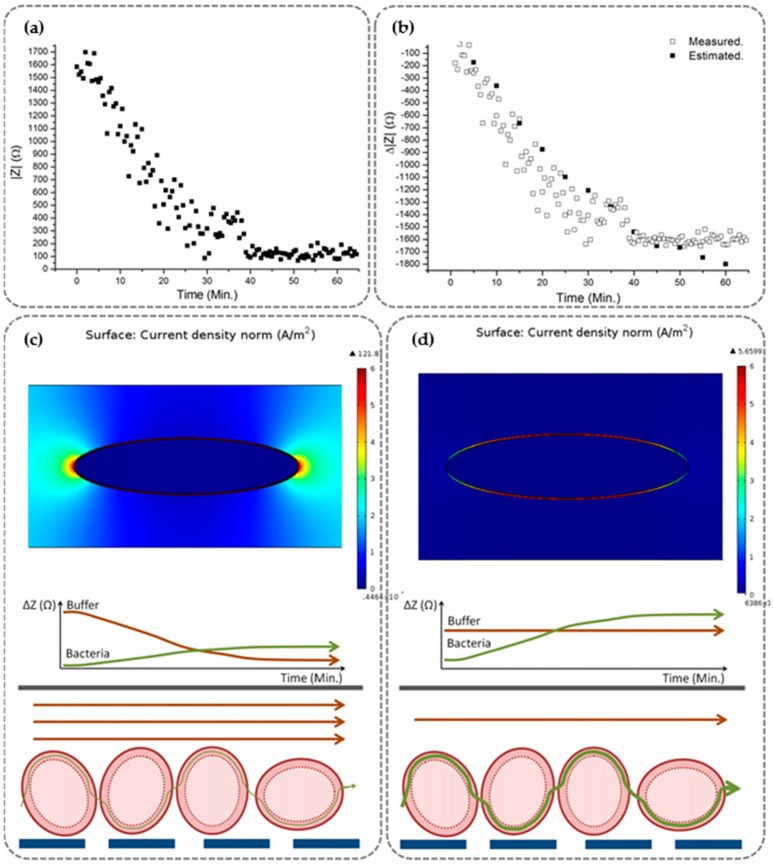
(**a**) Impedance magnitude; (**b**) Estimated versus experimental impedance; (**c**) Simulation of Comsol multiphysics of a single diluted cell on buffer of high conductivity steady buffer; (**d**) low-conductivity steady buffer. Flow path and influence to impedance quantification of both buffer conductivity and trapped bacteria (reproduced with permission from [36]).

The measured bio-impedance (|*Z*|), in Figure 6a, demonstrates that the impedance decreases and the concentration of trapped cells increases, without taking the frequency into account. Figure 6b shows the change of impedance (Δ|*Z*|) during the trapping course. 

This new optimized protocol enables an electrode multiplexing system that disables DEP voltage when the IA is enabled for concentration monitoring. Changes in sample conductivity dominate the bio-impedance measurements when left uncontrolled. With this approach, the surface current density of bacteria (Figure 6c,d) and the impedance is totally related to the conductivity from the sample buffer instead of the bacteria concentration (Figure 6c). Current density is principally placed at the cell membrane by controlling buffer conductivity (Figure 6d), and changes in impedance related to the quantity of trapped bacteria. Furthermore, including a bacteria-cleaning step in the protocol demonstrated an effective bio-impedance control of the resulted sample concentration in this study [36]. If applied, this last reviewed improvement could change the data of previous results. Moreover, all the improvements are a “must” to be considered in the development of new emerging devices.

## 4. Future Perspectives of DEP and IA On-Chip Platforms

Despite the numerous advances in DEP and IA systems for bacteria concentration and detection evidenced throughout this review, commercialization remains a daunting task to be addressed in the coming years. Currently, it is still challenging to find electronic devices combining electronics and microfluidics for a portable DEP system [41]. Regular commercial devices do not demonstrate a superior alternative required to replace current technologies [26]. Moreover, most of the microfluidic devices are limited to proof-of-concept and publications [19,135] due to the absence of consumer development and validation of market needs [135]. 

Because of the size of bacteria (most of them are 0.2 μm of diameter), miniaturization and automation of the complete system constitutes a challenge to be addressed [63,136,137,138]. Research for miniaturization is also driven by the need to reduce costs by, among other things, increasing throughput and automation [24]. Due to the current trend to develop fully-integrated lab-on-a-chip devices instead of bench-top devices [26], efforts need to be made to successfully integrate laboratory functions on single miniaturized chips as new emerging diagnostic devices [25]. Therefore the final product should be self-contained, not requiring prior sample treatment, preparation, or amplification [135,139].

Since microfluidic systems must contain some generic methods [19], many innovations are elaborated and difficult to fabricate. Therefore, the device requires labour intensive manufacturing techniques. The seamless integration of the different components will determine the portability, usability, simplicity of manufacturing and costs [135,139].

LOCs are considered the result of the convergence of chemical and biological analysis techniques and the engineering of computer chips [140,141]. This convergent scenario in areas such as micro-electronics, micro-sensors and bio-compatible materials makes possible the availability of cheaper and faster bio-devices [142]. It is in this context that there is a growing interest in fostering the cross-fertilization of Key Enabling Technologies (KETs), since these create value beyond the sum of the individual technologies for developing innovative and competitive products, goods and services [143,144,145].

Most of the microfluidic on-chip platforms for bacteria detection included in this work are the result of the convergence of KETs, namely, industrial biotechnology and micro- and nano-electronics. In particular, Nanotechnology is seen as one important KET for future diagnostics. An example is evidenced in the impact that nanospheres or nanoparticles can have in these devices [100,146]. In addition, it is expected that in the future, the convergence of other tangential KETs, such as Advanced Materials and Advanced Manufacturing Systems, could allow not only more effective and efficient analysis but also solve manufacturing and cost constraints. Therefore the key parameter to consider is industrialization, since production approaches always remain behind a new technology.

Even though there are pending challenges-opportunities, it is expected that point-of-care (POC) devices can generate $34.6 billion by 2021 on the global diagnostic market [147,148]. On the other hand, the market for microfluidics has been estimated to be $1.6 billion with a forecast rise to $3.6–5.7 billon by 2018 [135]. It is expected that the rise of POC testing could improve the accessibility to medical services and improve and facilitate healthcare programs [149]. Undoubtedly, the application of major interest for microelectromechanical devices is balanced towards medicine [150]. It is expected that in the coming years, there could be widespread use of LOC and POCs in food safety and medical diagnostics [151,152]. 

## 5. Technology Transfer and Social Return Challenges in Microelectronics

New emerging technological innovations such as those discussed in this review for bacteria concentration and detection should be assessed not only from a research perspective, but also taking into account a market-orientation view in order to foster innovation and successfully reach the final process of technology transfer, which is commercialization. Academics tend to focus their research on the proof-of-concept phase for a single-chip experiment (chip-to-chip or batch-to-batch) [135,139], therefore there is a conflict of interest between academia and market which results in reproducibility failures and LOC variabilities [139]. 

This concern has been addressed by the European Commission in recent years through their Framework Programme Horizon 2020, the financial initiative for research and innovation. Unlike previous funding initiatives, this is advocated to solve major societal challenges by overcoming the gap between research and market through the industrialization of previously mentioned KETs. 

Social availability and accessibility of these technologies is a little discussed topic. Bacteria diagnostic tests need to scope large populations; they will have more impact when everyone can use them [153,154]. In this sense, microfluidics should satisfy the needs of non-expert users so that it can become a routine operation for untrained personnel [19,139]. Moreover, market uncertainty is reduced if the product does not require new skill sets from consumers [155]. In particular, modelling and designing DEP and IA devices become critical for implementing systems for near-patient clinical analysis [41]. These devices would constitute an alternative of existing technologies, with minimal technological investment and allowing a higher level of market acceptance and uptake [139].

These technological innovations require the coordinated collaboration of researchers, through innovation communities, in order to overcome research-market barriers [156]. Since healthcare is a global process, knowledge-share activities require the continuous interaction of multiple actors [157]. Therefore, transferring knowledge from basic research to commercial organizations should be a responsibility from the universities, research centres, governmental bodies and the industrial sector [158], facilitating therefore shortest times-to-market [159].

**Figure 7:**
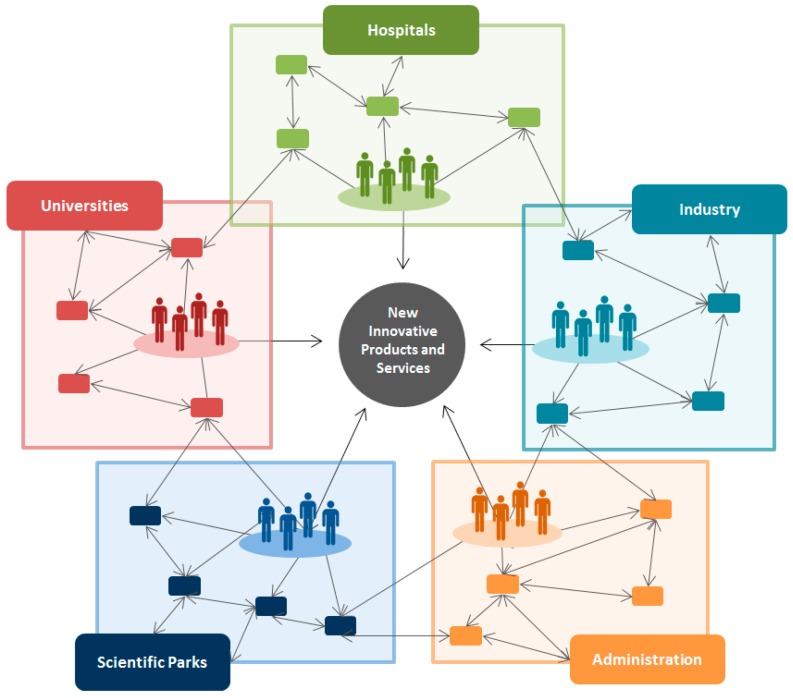
Scheme of a multidisciplinary ecosystem of stakeholders collaborating in the development of emergent devices (inspired from [144]).

In recent innovation models literature, there has emerged the “Five-Helix Model” concept [160,161] aimed at satisfying the needs of the healthcare system including life sciences such as medicine, biotechnology and the nanotechnologies. This concept emphasises the need of a coordinated cooperation among universities, hospitals, industry, administration and science parks (Figure 7). The schematic framework of this process resumes a multidisciplinary team, in the context of an innovative community ecosystem in which the resulting scenario can be the social return of public-funded investments.

## 6. Concluding Comments

In recent years, emerging microfluidic platforms combining dielectrophoretic and impedance analysis for bacteria concentration and detection have been developed for replacing conventional diagnosis techniques. These approaches respond to the need for more rapid, portable, simple and labour-saving bacteria-detection devices. Different research groups have demonstrated their feasibility by addressing different aspects. LoD and detection time, as well as sensitivity of devices have been modified during recent years. Some improved approaches include technical adaptations such as EP and AC-EO. In addition, several groups have developed enhancements in the combined system aimed at improving selectivity, detection times, conductivity variations and particle manipulation. 

It has been shown that selectivity could be improved by the use of antigen-antibody or fluorescent polystyrene beads, this last approach used in sporulated stages of bacteria. However, the costly and time-consuming difficulties of these labelled-based methods have resulted in other selectivity improvements such as the cDEP or iDEP, aimed at avoiding fouling by the use of a passivation layer. The introduction of Impedance Analysis strengthen the characteristics of a DEP-based devices, being a rapid, sensitive and accurate technological tool for bacteria concentration measurement, as well as a straightforward technological application of feedback between the device and a post-processing tool. This feedback allows the system to perform critical functions aiming for a rapid, accurate and selective device, such as the real-time interaction with the user, the automation of the process, and the implementation of intelligent algorithms to enhance its performance. As an example, conductivity variation correction, as it has been demonstrated by only one group of researchers, can be executed through a specially designed automated protocol. These approaches are the basis of new microfluidic platforms with other future challenges still to be addressed, for example, their miniaturization, automatization and commercialization by considering economies of scale, customer acceptance, market adoption, and what is also important: accessibility and social benefit. All of these perspectives cannot be accomplished without a collaborative ecosystem of multidisciplinary stakeholders able to transfer technological innovations by narrowing the gap between basic research and society.

## Figures and Tables

**Table 1:** Combined dielectrophoresis and dmpedance systems for bacteria concentration and detection.

Principle	Buffer	Conductivity	Bio-Affinity Element	Applied Frequency	Flow Rate Conditions	Bacteria	Sample Rate	Concentration	Signal Variation	Reference
DEP + IA	Manitol solution	0.2 mS/m	polyclonal antibodies	1 MHz	9 × 10^2^ μL/min	*E. coli* strain K12	NA	10^7^ cells/mL	NA	[38]
EPA-DEP + IA	DI water	0.2 mS/m	no element	100 kHz	5 × 10^2^ μL/min	*E. coli* strain K12	NA	10^4^ to 10^2^ CFU/mL	NA	[116]
iDEP + IA	DI water	1–2 μS/cm	fluorescent beads (2 μm)	100 Hz	40 μL/min	*B. subtilis* spores	10 μL/min	10^6^ spores/Ml	NA	[46]
nDEPpDEP + IA	Manitol solution	0.1 mS/m	no element	1 kHz (nDEP) and 100 kHz (pDEP)	0.27 m/s	*E. coli* strain K-12 (NBRC3301)	NA	NA	NA	[35]
pDEP + IA	PBS solution and DI water	low	polyclonal antibodies	100 Hz–1 MHz	2–4 μL/min	*E. coli* O157:H7	3 × 10^5^ CFU/mL	3 × 10^2^ CFU/mL	NA	[14]
DEP + IA	Milli-Q water	0.5 × 10^−3^ to 2.5 × 10^−3^ S/m	no element	500 Hz to 5 kHz	10 μL/min	*E. coli* 5K strains	NA	2 × 10^7^ cells/mL	3.1%	[36]
DEP + IA + (AC-EO)	Phosphate buffered saline (PBS at pH 7.4)	1.8 mS/m	no element	10 kHz–63 MHz (AC-EO)	5 μL/min	*S. epidermidis* ATCC 35984	NA	3.5 × 10^5^ CFU/mL and 3.8 × 10^6^ CFU/mL	NA	[37]
nDEP + IA	Drinking water	0.0086 S/m (aprox)	no element	1 kHz–10 MHz	25 μL/min	*E. coli* ATTC 8739	(150–1500 CFU/mL)	300 CFU/mL	1.13% ± 0.37%	[30]

DEP: dielectrophoresis; iDEP: insolator-based dielectrophoresis; pDEP: positive dielectrophoresis; nDEP: negative dielectrophoresis; IA: impedance analysis; EPA: electropermeabilization; AC-EO: AC electroosmosis; NA: No data available.

## Appendix

**Table A1:** Conventional bacteria concentration and detection methods.

Method	Type	Principle	Advantage	Limitation	Ref.
**Capillary Electrophoresis (CE)**	Electro-dynamic	Separation method based in sublimities capillaries and micro/nano fluidic changes	Technique that brings speed, quantifiability, reproducibility and automation	Long separation times, poor specificity, sensitivity of the analyte to the surrounding analytical environment, requirements for sample purity, and microbe aggregation. high salt buffers	[13]
**Mass Spectrometry (MS)**	Chemical Method	Identification of cells by breaking them into ionized molecular fragments and measuring mass/charge ratio of the products	Fast technique with high sensitivity, quantitative and qualitative analysis, differentiates isotopes	Lack of sample purity, chemical differences in cell species, variations between stages of cell development	[12]
**Centrifugation**	Physical Method	Separation technique based on the centrifugal force that separate particles in solution according to their size, shape, density, and viscosity	Rapid, inexpensive, simple, non-specific; amenable to large sample sizes	Bacteria adhere to and sediment with matrix components	[6]
**Filtration**	Physical Method	Mechanic force used to separate solids from fluids, liquids or gases by interposing a medium through which only the fluid can pass	Rapid, inexpensive, simple, non-specific; amenable to large sample sizes	Limited to low particulate foods that will not clog the filter and by the volume of sample that can be passed through the filter (i.e., sample filterability). Sample pre-treatment with enzymes and detergents can increase sample filterability but may adversely affect cell viability	[6]
**Immunoseparation**	Biological Method	Separation technique based the use of immunoglobulins (antibodies) reactive with the particles to be separated	rapid, simple, standards methods available	high-non-specific binding	[6]
**Raman microprobe spectroscopy (RMS)**	Microscopy	Spectroscopic fingerprint from the microbial sample. Provides quantitative and qualitative information that can be used to characterize, discriminate and identify micro-organisms at the single-cell level	High sensitivity and unique molecular specificity	The signal in direct aqueous solution detection is often weak because of the small polarizability of most biological molecules compared with dye probe molecules	[15,16]
**ELISA**	Immunologic	Use of antibodies to which enzymes have been covalently bound. The antigen is rapped so that it may be the target micro-organism or target toxin	Useful for detection of infectious and toxigenic bacteria (ex. C. perfringens a toxin in the intestinal contents of animals). Able to differentiate the e and b toxins	Is time-consuming, not very sensitive, and involves laborious multiple steps	[162]
**Polymerase Chain Reaction (PCR)**	Nucleic acid probe-based method	Is an in vitro technique, which allows the amplification of a specific DNA region that lies between two regions of a known DNA sequence	Rapidly detects a wide range of micro-organisms in foods, the environment and in biological material. Cheaper and robust technique	A major disadvantage is that the amount of DNA sequence known for a given organism may be limited	[18]
**Ligase chain reaction (LCR)**	Nucleic acid probe-based method	An in vitro nucleic acid amplification technique that exponentially amplifies targeted DNA sequences	Possesses unique advantages for sensitive and specific miRNA detection. LCR exhibits better specificity than primer extension-based amplification, such as PCR, RCA, LAMP	Limited by gel electrophoresis separation or heterogeneous analysis process, which brought about multiplex steps, high cost, and long analysis time	[17]
**Microarrays**	Nucleic acid method	Analysis of large numbers of genes at a high resolution by the hybridization of labelled DNA to a substrate containing thousands of surface-immobilised DNA’s or oligonucleotides	Micro-arrays allow thousands of specific DNA or RNA sequences to be detected simultaneously on a small glass or silica slide only 1–2 cm^2^ in size	Micro-array instruments are expensive, of limited availability and require much skill in extracting useful information from the plethora of available data. However, this is an exciting area that appears headed for a very bright future	[18]
